# The Book World of Medicine and Science

**Published:** 1901-11-02

**Authors:** 


### THE BOOK WORLD OF MEDICINE AND SCIENCE.

UTERINE TUMOURS: THEIR PATHOLOGY AND TREATMENT. By W. ROGER WILLIAMS, F.R.C.S. (London: Baillière, Tindall, and Cox. 1901. Price 10s. 6d. net.)

THIS book is blessed with the virtue of possessing an argument. The question is as regards the origin of neoplasms, not necessarily tumours of the uterus alone, and it runs thus:—Do neoplasms arise, as Johannes Müller believed, through a modification of the formation process; or are they the outcome of the inflammatory process, as Broussais maintained, owing to the intrusion of microbes or other irritants, *ab extra?* In other words are they of *intrinsic* or *extrinsic* origin? Mr. Roger Williams inclines to the former alternative, believing that neoplasms arise mainly from the play of forces generated within the body. He suggests that in a large proportion of cases the origin of neoplasms is associated with developmental irregularities, and he shows, in regard more particularly to cancer, that these growths are most prone to occur where cells which are still capable of growth and development most abound. As to the reason why such cells should take on such erratic growth as to determine the occurrence of cancer Mr. Roger Williams is entirely opposed to those who regard cancer as a disease of the poor. He says that during the last half century the wealth of the country has more than doubled, its pauperism has diminished more than one-half, wages have gone up, prices of most eatables have fallen, the consumption of meat per head has nearly doubled, yet the cancer mortality has more than quadrupled. "The only part of the United Kingdom where the cancer mortality has undergone no such marked increase is Ireland, which is just the one part where material progress has been deficient." Such considerations, he adds, seem to negative the doctrine of cancer being *morbus miseriæ*. They point to the greater prevalence of the disease among the well-to-do and easy-going, who habitually eat more than is good for them. In regard to the question of infectivity he says that there is no proof that this disease has ever been communicated from one individual to another, and he lays stress upon the fact that "there is not a single well-authenticated case on record" of cancer of the penis being acquired from intercourse with women the subjects of uterine cancer. On the other hand, he says that with regard to the auto-inoculability of cancer the evidence is so weighty as to be practically conclusive. Thus throughout the book we meet with many arguments and suggestions which go to support the main thesis of the author. It must not be imagined, however, that Mr. Roger Williams allows himself to be the slave of his argument, or that his book is a mere essay in support of a particular pathological thesis. In fact it is a very good practical account of the whole subject of uterine tumours, including their histology, their clinical symptoms, their pathology and their treatment. If we have referred more particularly to the underlying idea it is because it seems to be the *motif*, if one may so say, which has been running in the author's mind while the book has been in preparation.

A full and well illustrated account is given of the operative treatment of myomata and the question of the selection of cases is carefully discussed, for the author entirely dissents from the practice of some surgeons in removing these tumours whenever they are recognisable. If this practice were generally followed he calculates that "nearly a million operations of this kind might be done in a single year." "As the average mortality of such operations may be estimated," he says, "at about 10 per cent., this means that about 100,000 lives would be sacrificed in a single year; whereas, if the disease were left to its natural course . . . no more than 500 lives would be lost." This is a strong statement. In dealing with the treatment of cancer Mr. Roger Williams is at one with most modern surgeons in urging the necessity of early diagnosis and thoroughness of treatment, while his practical grasp of the whole subject is shown by the attention he bestows on "palliative operations." There is no doubt that much may be done by surgery to relieve suffering and make life endurable long after all hope of cure must be abandoned.

DISEASES OF THE ANUS AND RECTUM. By D. H. GOODSALL, F.R.C.S., and W. ERNEST MILES, F.R.C.S. In two parts: Part I. (London: Longmans, Green and Co. 1900. Price 7s. 6d. net.)

THIS book is a most careful study of a group of diseases which has we fear been very much neglected by many practitioners. There is, no doubt, a taste in these matters, and there are surgeons who greatly dislike to meddle with these complaints. The fact remains, however, that they are among the most painful and irksome of maladies, and that there are few things for which men are more anxious to obtain relief than even the most trivial diseases of the anus and rectum. The treatment of the whole subject by the authors of this book is thoroughly good. They have evidently observed well, and have applied their observations with skill and intelligence. The four diseases dealt with are abscess and fistula, fissure and piles. The description of the course and consequences of the different varieties of abscess is very good, and leads naturally to the large subject of fistula. In the treatment of abscess in this region the authors strongly recommend the employment of a T-shaped incision. They feel sure that the smallness of the opening so often made and the fact that it is linear, either radiating from or parallel to the anus, are largely responsible for the frequency with which abscess is followed by fistula. A T-shaped incision on the contrary, made, as they recommend, to extend beyond the limit of the area of inflammation, tends to gape even when no manual traction is being exerted upon its sides, and is but seldom followed by such a result. The portion of the book which deals with ano-rectal fistula is remarkably complete, and is a very instructive bit of work. Its main teaching may be summed up in two sentences: first, that every part of the fistula which can be got at without interfering with the internal sphincter is to be thoroughly laid open, and secondly, that above all things, the internal sphincter is not to be divided, even though for the sake of saving it an ascending branch of the fistula may have to be left unclosed. The details of the various kinds of fistnla are described with much care and minuteness. In regard to piles the treatment recommended is ligature. The matter is not to be discussed; ligature is the operation and there is an end of it, which makes the matter very simple. Thus the book is a guide to treatment rather than a complete essay on the subject; and a guide expects to be followed. Still it is an admirable handbook, careful, minute, and fully illustrated.

### BOOKS RECEIVED.

JOHN WRIGHT AND CO.

"Golden Rules for Diseases of Children." By George Carpenter, M.D.Lond., M.R.C.P. "'Golden Rules Series," No. xi.

BAILLIÈRE, TINDALL, AND COX.

